# High-quality and Large-size Topological Insulator Bi_2_Te_3_-Gold Saturable Absorber Mirror for Mode-Locking Fiber Laser

**DOI:** 10.1038/srep38444

**Published:** 2016-12-05

**Authors:** Hou-Ren Chen, Chih-Ya Tsai, Hsin-Ming Cheng, Kuei-Huei Lin, Po-Hsiu Yen, Chyong-Hua Chen, Wen-Feng Hsieh

**Affiliations:** 1 Department of Photonics and Institute of Electro-Optical Engineering, National Chiao Tung University, 1001, Ta-Hsueh Rd., Hsinchu, 30010, Taiwan; 2 Research Center for Applied Sciences, Academic Sinica, Taipei 11529, Taiwan; 3 Material and Chemical Research Laboratories, Industrial Technology Research Institute, Hsinchu 310, Taiwan; 4 Department of Applied Physics and Chemistry, University of Taipei, 1, Ai-Guo West Rd., Taipei, 100, Taiwan

## Abstract

A novel high-quality, large-size, reflection-type topological insulator Bi_2_Te_3_-Gold (BG) film-based nonlinear optical modulator has been successfully fabricated as a two-dimensional saturable absorber mirror (SAM) by pulsed laser deposition (PLD). This BG-SAM possesses saturation fluence of 108.3 μJ/cm^2^, modulation depth (Δ*R*) of 6.5%, non-saturable loss of 38.4%, high damage threshold above 1.354 mJ/cm^2^ and excellent uniformity providing for the generation of passive mode-locked (ML) pulses for erbium-doped fiber lasers (EDFLs) on a large sample area. Under 124 mW 976 nm pumping, We obtained 452-fs continuous-wave ML pulses with pulse energy of 91 pJ and full width at half-maximum (FWHM) of 6.72-nm from this EDFL. The results clearly evidence that the PLD is an efficient method for fabricating BG-SAM that is suitable for a compact ultrafast ML fiber laser system.

Ultrafast mode-locked fiber lasers (MLFLs) operating in the near infrared range have become one of the most active fields in laser research because of their wide and significant applications in optical communications^1^, industry, military and basic science. For passively MLFLs, a saturable absorber (SA) is indispensable in shaping the pulses in the temporal domain^2^. Among the various types of SAs, semiconductor saturable absorber mirror (SESAM)^1^,^3^, single-wall carbon nanotubes (SWCNTs)^4^,^5^,^6^,^7^,^8^, and graphene^9^,^10^,^11^,^12^,^13^ were intensively used in various MLFL schemes. Although SESAMs have been mostly used in laser resonators to generate mode-locked laser pulses, they are expensive, complicated in fabrication processes (e.g., grown on lattice-matched semiconductor substrates with high quality distributed Bragg reflector mirror), and narrow in wavelength tuning range (typically a few tens of nanometers)^1^. The carbon nanotubes (CNTs) have been considered as excellent SAs for its easy fabrication and low-cost, but their operation wavelength is determined by the nanotube diameters and chirality^14^. The broadband (>300 nm) CNT-SAs can be made in use of different diameters and chiralities of nanotubes^5^, but usually leads to a larger non-saturable loss. Although graphene possesses the broadband wavelength-independent saturable absorption ranging from approximately 0.8 to 2.5 μm^9^,^10^, it has a small absorption of only 2.3% and a low modulation depth of ~0.75% at 1060 nm. The small modulation depth might not be enough to suppress continuous-wave (CW) components to obtain mode-locked pulses in high gain laser systems (e.g., Yb-doped fiber laser system). Therefore, much attention has been paid to develop new SAs, which are expected to have the characteristics of broadband-wavelength saturable absorption, low saturation intensity, large modulation depth, high damage threshold, and low cost. Most recently, a newly discovered state of quantum matter, graphene-like two-dimensional (2-D) materials, namely topological insulators (TIs) such as Bi_2_Te_3_, Bi_2_Se_3_, and Sb_2_Te_3_, have been extensively developed. Those TIs have typical characteristics with a small insulating bulk gap of 0.2–0.3 eV, corresponding to broadband nonlinear optical response from visible to 4.1 μm^15^ and gapless metallic state in the edge/surface around the Γ point^16^,^17^. Noteworthy, TIs own unique electronic and optical properties^16^ having good thermal management, ultra-broadband saturable absorption features and giant third-order optical nonlinearity^18^,^19^,^20^. Promising results show that TIs are good candidates as saturable absorbers for generating short and high-energy laser pulses.

In 2012, Bernard *et al*. first reported that Bi_2_Te_3_ exhibits SA behavior at 1550-nm wavelength range^21^. Afterward, with the advantages of the saturable absorption behavior of TI-based SAs, Zhao *et al*. further demonstrated a passively mode-locked ultrafast fiber laser by inserting the TI-SAs into the laser cavity^20^,^22^. Since then, TIs, which were manufactured with various methods such as mechanical exfoliation method^23^,^24^, bulk-structured TI method^25^,^26^,^27^,^28^, and wet chemical synthesis method (e.g., polyol method^20^,^22^,^29^ and hydrothermal intercalation with liquid phase exfoliation method^30^,^31^,^32^,^33^,^34^,^35^), have been successfully used as the SAs for generating ultrafast ML laser pulses. Although the mechanical exfoliation and bulk-structured TI methods can obtain low-cost and high-quality TI films by using scotch tape to repeatedly peel the film from the surface of the bulk crystal, it has the lowest repeatability. Furthermore, the wet chemical synthesis method is widely applied to obtain low-cost few-layer nanomaterials that are usually doped into polyvinyl acetate (PVA) to form TI/PVA film^36^,^37^ and to be sandwiched between two fiber ferrules in laser cavity. However, the PVA-based SAs have less uniformity and are less liable to optical damage by high intracavity power and mechanical damage by fiber connectors. Recently, TI was deposited on the side-polished fiber (SPF) and microfiber to sustain high-power laser operation. Those devices exploit the interaction of the intracavity light evanescent field with TI materials. For bulk-structured TI method, the TI must be polished smooth, then attached on the SPF^25^,^26^. A small amount of index-matching oil is required to spread on the flat surface to induce the evanescent wave interaction with TI layer. Hence, the bulk-structured TI-SPF device is not suitable for package in the compact laser system. Boguslawski *et al*.^38^,^39^ directly deposited the Sb_2_Te_3_ film on SPF by means of a single planar magnetron that makes the TI film tightly coupled with surface of SPF, therefore, it is not necessary to drop the index-matching oil. However, the non-symmetrical geometry of the SPF-SAs made of deposition 1 mm long TI film induces the polarization dependent losses (PDL) of ~2.7 to 5 dB^38^,^39^ and even larger PDL of ~17 dB had been obtained by depositing 5 mm long TI film^39^. In addition, it isn’t easy to control the polishing parameters of SPF, the distance between core boundary and polished surface, polishing length, and roughness of polishing surface, which highly affect the interaction strength of evanescent field with TIs and insertion loss (scattering loss). The microfiber needs a small cladding diameter (<50 μm) to enhance the interaction strength of the evanescent field with SAs^40^,^41^; therefore, the optical property of TI-microfiber SAs is strongly dependent on the diameter of microfiber. However, it is difficult to repeat the fabrication process and the SAs are prone to fracture. As it is well-known, PLD is a mature approach to obtain thin film by focusing the high energy laser beam onto the target in vacuum chamber and depositing the vaporized plasma plume on the substrate. In comparison with the molecular beam epitaxy (MBE)^42^, it has a relatively low-cost target, low growth temperature and doesn’t require to operate in ultra-high vacuum environment. The film thickness can be easily controlled by changing the deposition parameters, including laser pulse energy, deposition temperature, and processing time. Many investigations have shown that TIs presents interesting van der Waals epitaxial growth behavior on single-crystalline substrate and amorphous surface such as SiO_2_ substrate^43^, mica substrate^44^, and Al_2_O_3_ substrate^45^. Therefore, it has the potential to grow TIs on any substrate including soft-substrate for saturable absorption applications.

In order to cost-effectively fabricate TI-based SAs with excellent uniformity, repetitiveness, simplicity, in this work, a new-type high-quality and large-size BG-SAM with the above-mentioned merits have been grown on *c*-Al_2_O_3_ substrate by PLD. The BG-SAM exhibits the saturable absorption characteristics, identified by nonlinear reflectivity measurement. We obtained the saturable fluence, modulation depth (Δ*R*), non-saturable loss, and damage threshold of BG-SAM to be 108.3 μJ/cm^2^, 6.5%, and 38.4%, and 1.354 mJ/cm^2^, respectively. The BG-SAM not only enables the generation of 452.3 fs soltion pulses with 20.1 MHz repetition rate, 6.72-nm FWHM, and 1.82-mW output power, but also capable of generating ML pulses on an ultra-large sample area. Our results provide a practical photonic device which is suitable for application in the compact ML fiber laser systems.

## Results

### Structural and optical analyses of Bi_2_Te_3_-Gold saturable absorber mirror

**Figure 1:**
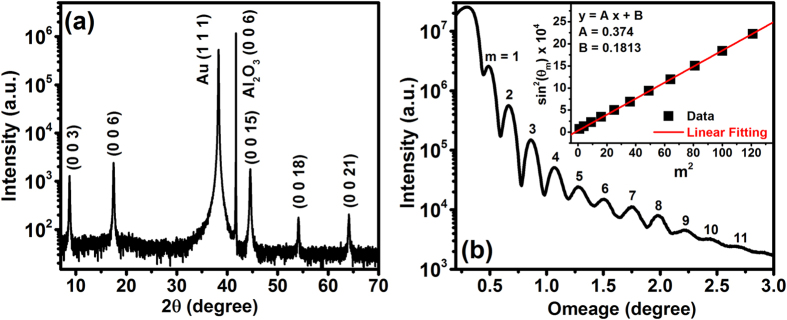
(**a**) XRD measurements on a BG-SAM sample showing only the (0 0 3) family of Bi_2_Te_3_ diffraction peaks. (**b**) XRR curve of Bi_2_Te_3_ coated on a *c*-Al_2_O_3_ substrate. Inset shows the fitted XRR data based on the modified Bragg equation.

Figure 1(a) exemplifies the 2*θ-*ω X-ray diffraction (XRD) pattern for Bi_2_Te_3_-Gold thin film on *c*-Al_2_O_3_. The XRD shows only the (0 0 6) of *c*-Al_2_O_3_ substrate, (1 1 1) of Gold, and (0 0 3) family of Bi_2_Te_3_ diffraction peaks that verifies the Bi_2_Te_3_ film is well aligned along *c*-axis. A result of X-ray reflectivity (XRR) analysis on the BT thin film is presented in Fig. 1(b), in which the steeply decaying XRR curve is modulated by several oscillation peaks known as the Kiessig fringes. Film thickness derived from the frequency of the Kiessig fringes is fitted to a modified Bragg equation^46^:


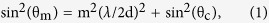


where *θ*_*m*_ is the maximum of the *m*-th oscillation peak of the Kiessig fringes starting from the low end *θ*, and *d* is the film thickness. Therefore, the thickness *d* of the Bi_2_Te_3_ film can be estimated by linear fitting from the angular separation between the oscillation maxima, according to the modified Bragg equation using the first to the eleventh oscillation maxima as shown in the inset of Fig. 1(b). The estimated thickness of Bi_2_Te_3_ film is about 18 nm.

**Figure 2:**
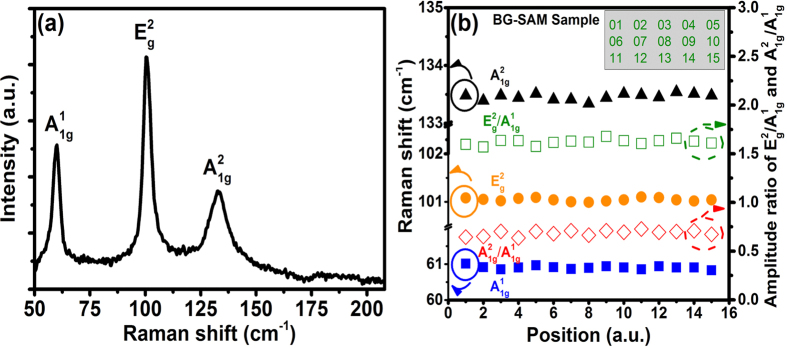
(**a**) Raman spectra of Bi_2_Te_3_ on Gold/*c*-Al_2_O_3_. (**b**) The amplitude ratio (normalized to A^1^_1g_ peak) and Raman shift at 15 different BG-SAM positions. The inset shows the measured relative positions on the sample.

Figure 2(a) shows three characteristic Raman peaks of BG-SAM at 60.9, 101.1, and 133.4 cm^−1^, which are consistent with the A^1^_1g_, E^2^_g_, and A^2^_1g_ vibrational modes of BT film^47^,^48^,^49^, respectively. In order to evaluate the quality and uniformity of BG-SAM (sample size: ~20 × 15 mm^2^), we collected the Raman spectra sequentially from left to right and top to bottom with total 15 different positions on the sample (inset of Fig. 2(b)), and the measured results of phonon frequencies and amplitude ratio (normalized to A^1^_1g_ peak) were shown in Fig. 2(b). The average amplitude ratio of E^2^_g_/A^1^_1g_, A^2^_1g_/A^1^_1g_, and phonon frequencies for A^2^_1g_, E^2^_g_, and A^2^_1g_ are 1.62 ± 0.054, 0.686 ± 0.046, 60.9 ± 0.093, 101.05 ± 0.05, and 133.46 ± 0.1, respectively. The measured data has a small variation at different positions on the sample, indicating that our BG-SAM has high quality and excellent uniformity.

**Figure 3:**
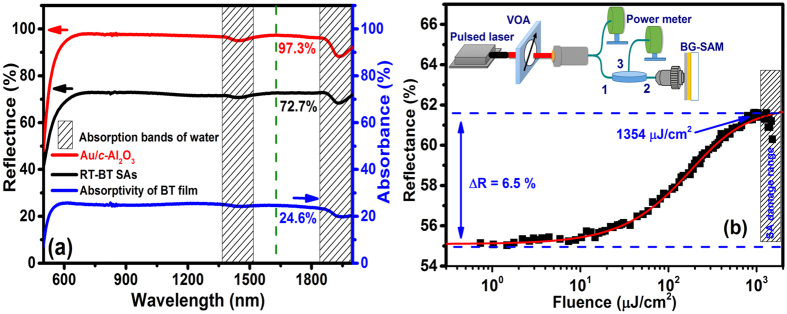
(**a**) Absorbance of the Bi_2_Te_3_ film derived from the reflectance spectrum of BG-SAM subtracting from that of Gold/*c*-Al_2_O_3_. (**b**) Nonlinear reflectance curve of BG-SAM. Dots: the measured data; solid curve: fitting to the data. The inset shows the schematic diagram of nonlinear reflectivity measurement setup.

Figure 3(a) illustrates linear reflectivity spectra of Gold/*c*-Al_2_O_3_ and BG-SAM. The average reflectivity of Gold film on *c*-Al_2_O_3_ is up to 97% from wavelength of 630 to 1800 nm (red curve) and ~72.7% for the BG-SAM (black curve). By subtracting the former spectrum to the latter one as the blue curve, we obtained a relatively broad and flat absorption characteristic from the 580 to 2000 nm for the Bi_2_Te_3_ film with average absorbance of to be ~24.6%, due to its Dirac-cone-like zero bandgap from the visible to mid-IR wavelength range, which is similar to ref. 36. This makes Bi_2_Te_3_ material usable for laser mode-locking applications in an ultra-broadband wavelength range. Since intraband and interband relaxation times of TIs are found to be more than 0.5 ps and 5 ps, respectively, they belong to slow SAs^50^,^51^,^52^, nevertheless, in soliton lasers the recovery time does not directly affect the pulse duration, which is determined instead by a balance between anomalous dispersion and nonlinearity in the cavity^53^.

Because the reflectance and modulation depth Δ*R* of BG-SAM highly depend on the alignment, e.g., contact angle and distance of the BG-SAM to the FC/PC connector. In order to make sure the measured nonlinear absorption parameters of the BG-SAM correspond to those for the CW-ML, we first carefully adjusted the contact angle between the BG-SAM and the FC/PC connector and made sure that CW-ML occurred at the low pumped power, indicating the coupling efficiency is high. Then, we turned off the pumped laser and injected the femtosecond laser to measure the nonlinear reflectance of BG-SAM under the incident fluence tuning from 1 to 1500 μJ/cm^2^ (limited by the available pump source). This procedure was repeated to obtain the almost same curves at different sample positions. A typical nonlinear reflectance curve is shown in Fig. 3(b), in which the nonsaturated (linear) reflectance is ~55.1% measured at low incident fluence. It is smaller than the average reflectance of ~72.7% in Fig. 3(a) measured by using an integration sphere. The smaller linear reflectance should result from the insertion loss of circulator and the coupling loss (~1.2 dB).

For slow SAs, the nonlinear parameters can be obtained by fitting the measured nonlinear reflectivity data to a theoretical model^54^,^55^, expressed as:


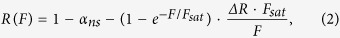


where *F* is the pulse fluence, *α*_*ns*_ stands for the non-saturable loss, Δ*R* is the modulation depth, and *F*_*sat*_ is the saturation fluence. We obtained the BG-SAM parameters of *α*_*ns*_ = 38.4%, *F*_*sat*_ = 108.3 μJ/cm^−2^, Δ*R* = 6.5% and damage threshold above 1.354 mJ/cm^2^. Although the BG-SAM needs a circulator in ring cavity, it has excellent uniformity, repetitiveness, simplicity over the SPF-SAs and the damage threshold of ~1.354 mJ/cm^2^ is three times larger than 460 μJ/cm^2^ for the transmission-type graphene/PMMA SAs^56^. The calculated saturation intensity *I*_*sat*_, using *I*_*sat*_ = *F*_*sat*_/*τ*_*A*_ with the recovery time of SAs *τ*_*A*_ = 5.7 ps from ref. 51, is about 19 MW/cm^2^, which is comparable with *I*_*sat*_ = 26.7 MW/cm^2^ for the Bi_2_Te_3_ film deposited on microfiber by PLD^57^ but it is much less than 180 MW/cm^2^ and 4.6 GW/cm^2^ for Bi_2_Te_3_-nanosheets and Bi_2_Te_3_-nanoplates in refs 36 and 37, respectively. The large variation of saturation intensity could be due to that they are strongly dependent on the morphology and size (surface-to-volume ratio) of the Bi_2_Te_3_^37^,^58^.

### Stable passive mode-locking of EDFL with Bi_2_Te_3_-Gold saturable absorber mirror

**Figure 4:**
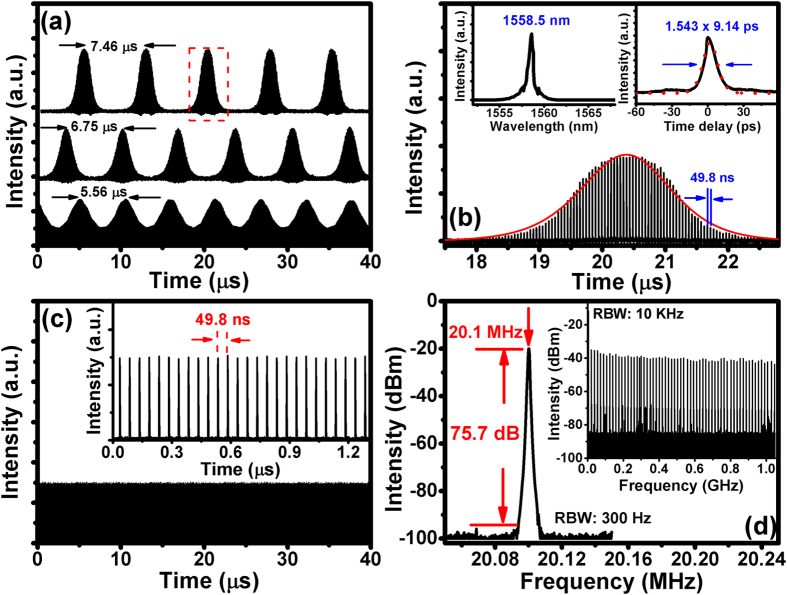
(**a**) QS-ML pulse trains at three operation conditions, corresponding to repetition rate of the QS envelope for 134, 148.4, and 179.9 kHz. (**b**) Expanded temporal shape of a single QS-ML pulse. The right inset shows the measured autocorrelation trace (black curve) and fitting curve by Sech^2^ function (red dotted curve). The left inset shows the corresponding optical spectrum. (**c**) Pulse train of the CW-ML laser with BG-SAM, as recorded with 0.15 μs/div and 5 μs/div (inset). (**d**) RF spectrum of MLFL (first beat note). Inset: the RF spectrum for 1 GHz scanning range.

The threshold pump power for continuous-wave lasing was approximately 69 mW. When the pump power is increased to 82 mW, the Q switched mode-locking (QS-ML) state can be observed. Figure 4(a) shows the measured results of output pulse trains in the QS-ML state by adjusting the contact angle between SA and FC/PC connector. The QS envelopes are very stable and appear repeatedly. The repetition rates for the QS envelope were about 134, 148.1 and 179.9 KHz with the output power of 0.51, 0.54, and 0.59 mW. Figure 4(b) shows the single temporal shape of a QS-ML pulse which is expanded from the red rectangle of Fig. 4(a). By fitting the envelopes with the bi-exponential formula^59^:


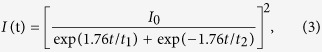


where *I*_*0*_ is the scaling factor, the rising time *t*_*1*_ = 1.81 μs and the falling time *t*_*2*_ = 1.99 μs of the temporal QS envelope can be obtained. The width of QS envelope *τ* = (*t*_*1*_ + *t*_*2*_)/2 = 1.90 μs. The right inset of Fig. 4(b) shows the width of the individual ML pulse inside QS envelope, measured by an autocorrelator (black curve, accumulating many QS envelopes), is about 9.14 ps determined by fitting to the Sech^2^ shape (red dots). The central wavelength of the QS-ML pulses is 1558.5 nm with FWHM of 0.85 nm, as shown in the left inset of Fig. 4(b). Therefore, the envelope width of the QS-ML, and pulse width of ML pulses were 1.99 μs, 1.71 μs, 2.66 μs, and 9.14 ps, 7.63 ps, and 3.26 ps, corresponding to repetition rates of 134, 148.1 and 179.9 KHz in Fig. 4(a), respectively. Except for 2.66 μs one, the results reveal the repetition rates of QS envelope increases, and envelope width of the QS-ML pulses and pulse width of ML pulses decreases as increasing the coupling efficiency (increasing intracavity power), which are inherent characteristics^3^,^8^. Since the 2.66 μs QS-ML is in the intermediate state to completely ML state against the QS-ML, the QS modulation becomes weakened^35^. By taking a close-up view of the ML pulses as shown in Fig. 4(b), the pulse-to-pulse separation was measured to be 49.8 ns, corresponding to fundamental frequency of 20.1 MHz, which matched with the cavity round-trip time. The loss from misalignment between SAs and FC/PC connector results in the difficulty for self-starting from QS instability into continuous-wave mode-locking (CW-ML) operation^6^,^60^. After carefully adjusting the contact angle, stable CW-ML occurs at increased output power of 0.65 mW. Once the stable CW-ML state occurs, the CW-ML can always self-start by raising the pumped power across the CW-ML threshold from the QS-ML state. Figure 4(c) shows the pulse train with a long time scale, revealing the CW-ML state is free from Q-switching modulation at 124 mW pumping. The inset of Fig. 4(c) shows a typical CW-ML pulse train with pulse spacing of approximately 49.8 ns, corresponding to fundamental repetition rate of 20.1 MHz, which agrees with the radio-frequency (RF) spectrum in Fig. 4(d). The RF spectrum shows the fundamental beating at 20.1 MHz with a very high extinction ratio of 75.7 dB against noise. The stable mode-locking operation was indicated by steady broad RF spectrum of harmonics measured up to 1 GHz as shown in the inset of Fig. 4(d).

**Figure 5:**
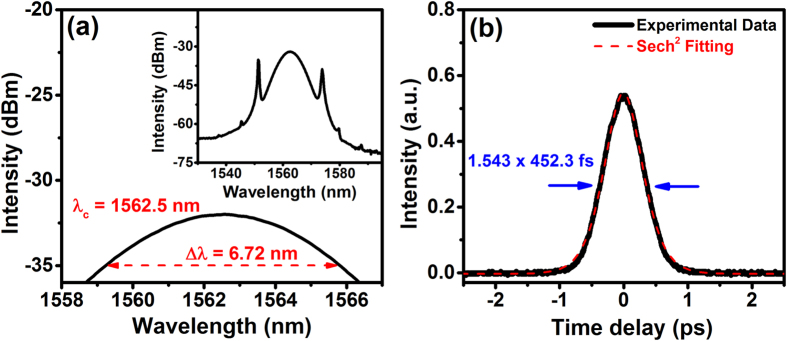
(**a**) The output optical spectrum of the mode-locked laser pulses. (**b**) The measured autocorrelation trace of the mode-locked pulses (solid line) and the fitting result obtained using a sech^2^ function (dotted line).

The inset of Fig. 5(a) shows the optical spectrum of MLFL at pump power of 124 mW. The laser peak is centered at 1562.5 nm with 6.72-nm FWHM and clear Kelly sidebands are observed on the optical spectrum, indicating that the mode-locked operation is operated in soliton regime. We also measured the pulse duration of ML pulses by using an autocorrelator as shown in Fig. 5(b). By fitting the autocorrelation trace to a sech^2^ function, we obtain the pulse duration to be 452.3 fs having the time-bandwidth product, TBP = 0.373. Due to high-order nonlinear effect, further increase of the pump power (>130 mW) results in wave breaking, and the laser would turn into multi-pulse states. Therefore, the maximum measured output power for single ML pulse state is approximately 1.82 mW with pump power of 124 mW. We estimated the pulse energy and peak power to be 91 pJ and 201 W, respectively.

**Figure 6:**
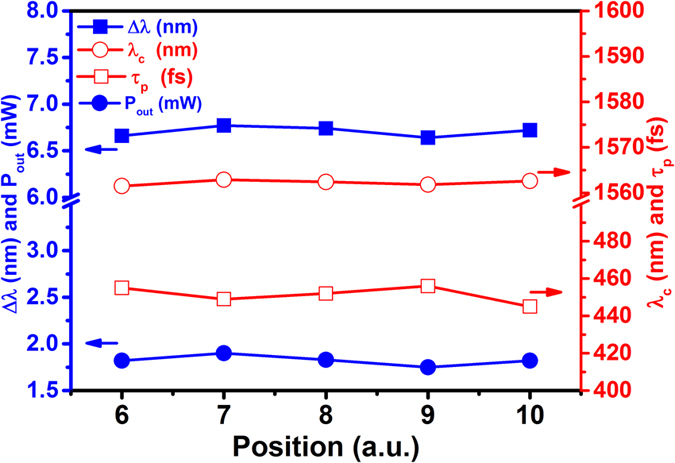
The center wavelength (*λ*_*c*_), FWHM of optical spectrum (Δ*λ*), ML pulse duration (*τ*_*p*_), and output power (P_out_) of EDFL operated on different positions of BG-SAM.

In order to investigate the quality of ML pulses operated on different positions of the BG-SAM, we move the BG-SAM along the positions with numbers 06–10 as shown in the inset of Fig. 2(b). The center wavelength (*λ*_*c*_), FWHM of optical spectrum (Δ*λ*), ML pulse duration (*τ*_*p*_), and output power (P_out_) at pumping power of 124 mW are recorded in Fig. 6. The average values *λ*_*c*_, Δ*λ, τ*_*p*_, and P_out_ over the 5 different positions are 1562.2 ± 0.7 nm, 6.706 ± 0.065 nm, 451.4 ± 5.5 fs, and 1.82 ± 0.075 mW, respectively. The results demonstrated that our BG-SAM allowed us to generate the ML pulses on different positions of sample due to its excellent uniformity. In this work, the BG-SAM mode-locked EDFL can be continuously operated for more than 24 hours.

## Discussion

For the first time, a high-quality, large-size, and ultra-broadband absorption BG-SAM for ML EDFL has been experimentally demonstrated. In contrast to TI SAs manufactured by mechanical exfoliation method, bulk-structured TI method, and chemical synthesis method, the BG-SAMs fabricated in this work possess excellent uniformity and fabrication repeatability, evidenced by the fact that the amplitude ratio and peak position of Raman shift are almost same and allowed for stable mode-locking of EDFL operated with different positions on the sample. We don’t observe any signs of self-mode-locking under identical conditions without the BG-SAM. The saturable fluence, Δ*R*, non-saturable loss, and damage threshold of BG-SAM are 108.3 μJ/cm^2^, 6.5%, 38.4%, and 1.354 mJ/cm^2^, respectively. The large modulation depth, which is critical for shortening the pulse duration, makes the BG-SAM more suitable for mode-locking high-gain fiber lasers. The passively mode-locked EDFL with pulse duration of 452.3 fs, FWHM of 6.72 nm, and output power of 1.82 mW at pumping power of 124 mW were obtained. We estimated the pulse energy and peak power to be 91 pJ and 201 W, respectively. Finally, the mode-locked pulse can be obtained at 5 different positions, indicating its excellent uniformity. In addition, BG-SAM can also be further cut into smaller piece and fixed on the end of FC/PC fiber connector like commercial SESAM. Our results provide a practical photonic device suitable for compact mode-locked fiber laser systems.

## Methods

### Fabrication of the Bi_2_Te_3_-Gold saturable absorber mirror

**Figure 7:**
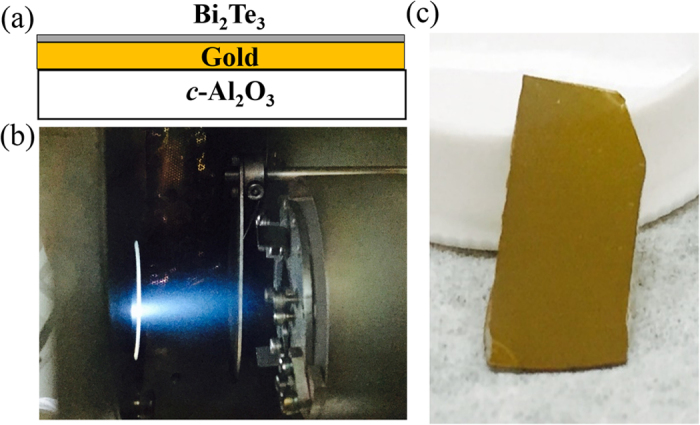
(**a**) Structure of Bi_2_Te_3_-Gold film on *c*-Al_2_O_3_ substrate. (**b**) Photograph of the PLD plasma plume while growing Bi_2_Te_3_ film, and (**c**) photograph of BG-SAM.

A schematic of Bi_2_Te_3_-Gold thin film is shown in Fig. 7(a), which is grown on a double-polished *c*-Al_2_O_3_ substrate by pulsed laser deposition (PLD) using a KrF laser (λ = 248 nm) with a high purity (5 N) Bi_2_Te_3_ and Gold targets. Before deposition, the *c*-A1_2_O_3_ substrate was cut into an area of 20 × 15 mm^2^ and cleaned using acetone, methanol, and de-ionized water in an ultrasonic cleaner for 15 minutes to remove organic contaminants. The target-to-substrate distance was set about 40 mm. The base pressure remained better than 1 × 10^−6^ Torr. First, Gold film was deposited on the *c*-A1_2_O_3_ substrate as the high reflectivity mirror at a substrate temperature of 50 °C. Then, the Bi_2_Te_3_ plasma plume (Fig. 7(b)) was deposited at a substrate temperature of 250 °C on Gold/*c*-A1_2_O_3_. Deposition was carried out using 1200 KrF laser pulses, with pulse repetition rate of 1 Hz. The photography of BG-SAM is shown in Fig. 7(c). Furthermore, Raman spectroscopy was used to confirm the quality of the BG-SAM under 532-nm excitation with a laser power of ~0.5 mW. The linear optical properties of BG-SAM have been investigated by using UV-visible-NIR spectrophotometer (Hitachi U4100). In addition, the structure of the Bi_2_Te_3_-Gold on *c*-Al_2_O_3_ is characterized by X-ray diffraction (XRD) 2*θ-ω* scans using a PANalytical Empyrean X-ray diffractometer (Cu K_*α*1_, *λ* = 1.54056 Å) to examine the out-of-plane orientation.

In order to determine the modulation depth, the nonlinear optical parameters of the BG-SAM were measured in a twin-detector power-dependent reflection measurement setup as shown in the inset of Fig. 3(b). A maximum output power of 100 mW, 1565-nm pulsed laser with 1-ps pulse width and 78-MHz repetition rate was used as the excitation source. A variable attenuator comprises a half-wave plate and a polarization beam splitter was used to adjust the pulse fluence on BG-SAM. A typical fiber coupler splits the pulse power into the reference and measurement arms. The back reflected power is measured through the circulator from the SA (measurement arm). At the end, the power in both arms is measured by the dual-channel power meter.

### Architecture of the mode-locked EDFL

**Figure 8:**
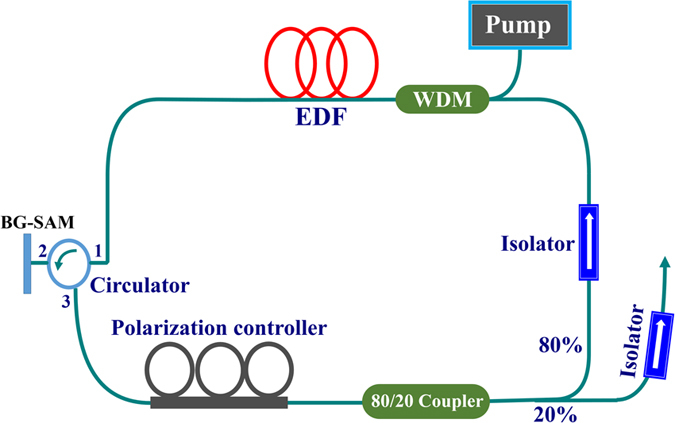
Schematic setup of the BG-SAM based mode-locked Er-doped fiber laser.

Figure 8 shows the schematic of our MLFL with the Bi_2_Te_3_-Gold film as the SA. The MLFL includes a piece of 1.1 m-long erbium-doped gain fiber (Er80-4/125, LIEKKI, *β*_2_ = 0.0593 ps^2^/m), and 8.9 m single-mode fiber (*β*_2_ = −0.023 ps^2^/m), corresponding to the net cavity dispersion of −0.139 ps^2^. A diode laser with a center wavelength of 976 nm was used to pump the erbium-doped gain fiber through a 980/1550 nm wavelength-division multiplexing coupler. A 20/80 fused fiber coupler was used to tap out the mode-locked pulses from the ring resonator with 20% output ratio. A polarization-independent three-port circulator was used to ensure the unidirectional operation, and to incorporate BG-SAM into the laser cavity. The BG-SAM was fixed on the mirror mount on a three-axis translational stage. The monitoring of the output pulse trains was performed with a high-speed InGaAs detector, which was used to convert the optical signal into electric signal, and then output to an oscilloscope (Leroy LT372, 500-MHz bandwidth). A power meter, an optical spectrum analyzer (Ando AQ6315A), and a radio frequency (RF) spectrum analyzer (HP 8560E) were used to measure the power, optical spectrum, and longitudinal mode beating, respectively. A non-collinear autocorrelator (FR-103WS, Femtochrome Research, Inc.) was used to measure the pulse duration.

## Additional Information

**How to cite this article**: Chen, H.-R. *et al*. High-quality and Large-size Topological Insulator Bi_2_Te_3_-Gold Saturable Absorber Mirror for Mode-Locking Fiber Laser. *Sci. Rep.* **6**, 38444; doi: 10.1038/srep38444 (2016).

**Publisher's note:** Springer Nature remains neutral with regard to jurisdictional claims in published maps and institutional affiliations.
